# The arginine transporter Can1 negatively regulates biofilm formation in yeasts

**DOI:** 10.3389/fmicb.2024.1419530

**Published:** 2024-06-05

**Authors:** Akira Nishimura, Ryoya Tanahashi, Kazuki Nakagami, Yuto Morioka, Hiroshi Takagi

**Affiliations:** ^1^Institute for Research Initiatives, Nara Institute of Science and Technology, Ikoma, Nara, Japan; ^2^Department of Food Science and Technology, University of California, Davis, Davis, CA, United States; ^3^Division of Biological Science, Graduate School of Science and Technology, Nara Institute of Science and Technology, Ikoma, Nara, Japan

**Keywords:** arginine transporter, biofilm, *Candida glabrata*, flocculation, *Saccharomyces cerevisiae*, virulence

## Abstract

The arginine transporter Can1 is a multifunctional protein of the conventional yeast *Saccharomyces cerevisiae*. Apart from facilitating arginine uptake, Can1 plays a pivotal role in regulating proline metabolism and maintaining cellular redox balance. Here, we report a novel function of Can1 in the control of yeast biofilm formation. First, the *S. cerevisiae CAN1* gene knockout strain displayed a significant growth delay compared to the wild-type strain. Our genetic screening revealed that the slow growth of the *CAN1* knockout strain is rescued by a functional deficiency of the *FLO8* gene, which encodes the master transcription factor associated with biofilm formation, indicating that Can1 is involved in biofilm formation. Intriguingly, the *CAN1* knockout strain promoted the Flo11-dependent aggregation, leading to higher biofilm formation. Furthermore, the *CAN1* knockout strain of the pathogenic yeast *Candida glabrata* exhibited slower growth and higher biofilm formation, similar to *S. cerevisiae*. More importantly, the *C. glabrata CAN1* gene knockout strain showed severe toxicity to macrophage-like cells and nematodes. The present results could help to elucidate both the molecular mechanism underlying yeast biofilm formation and the role it plays. Future investigations may offer insights that contribute to development of antibiofilm agents.

## Introduction

Arginine is a multifunctional amino acid in living organisms. In addition to being a proteogenic amino acid, arginine functions as a stress protectant: that is, it acts as an osmolyte, an oxidative stress protectant, a protein-folding chaperone, and a membrane stabilizer (Baynes et al., 2005; Nishimura et al., 2010; Mohan et al., 2012; Kim et al., 2016). Furthermore, nitric oxide synthesized from arginine plays a role in the tolerance to high-temperature and drought as well as in the regulation of sugar metabolism (Nishimura et al., 2013; Nasuno et al., 2014; Yoshikawa et al., 2016; Shino et al., 2022). In short, arginine is essential in maintaining cellular homeostasis by protecting cells from various environmental stresses. Therefore, arginine uptake and metabolism are vital for the survival of microorganisms.

In the yeast *Saccharomyces cerevisiae*, pathways for intracellular uptake of arginine include the arginine transporter Can1, its paralog Alp1, and, to some extent, the general amino acid transporter Gap1 (Grenson et al., 1970; Sychrova and Chevallier, 1994). Until recently, Can1 was considered to be the main arginine uptake protein in *S. cerevisiae* because it is the primary transporter of a toxic arginine analog, canavanine. However, despite the disruption of *CAN1*, arginine uptake activity does not decrease significantly, which raises questions about the specific function of Can1 in arginine uptake (Zhang et al., 2016; Nishimura et al., 2022). Several research groups, including ours, have reported additional functions of Can1 beyond arginine uptake activity. Our studies revealed that Can1 is a receptor (also known as a transceptor) for extracellular basic amino acids (arginine, lysine, and ornithine) and directly activates the protein kinase A (PKA) pathway without any increase in the cAMP level (Nishimura et al., 2020a,b, 2023a; Tanahashi et al., 2022, 2023). This mechanism is crucial for inhibiting proline utilization, although the further details remain unclear. Moreover, Can1 is known to be part of a microdomain structure on the cell membrane called the membrane compartment occupied by Can1 (MCC)/eisosome. MCC/eisosome functions as a sensor for environmental changes and cell membrane damage, suggesting that Can1 is involved in external environmental sensing (Lanze et al., 2020). Can1 also may contribute to extension of cellular lifespan by controlling redox homeostasis and endoplasmic reticulum stress response (Beaupere et al., 2017). Thus, Can1 is a multifunctional protein that may be extremely important for maintaining cellular homeostasis.

Biofilms are formed by aggregating microorganisms into multicellular structures that adhere to surfaces. Biofilm formation has been reported in both eukaryotes and prokaryotes. Biofilms are well known to bind tightly to various surfaces, including those of plastics and cells, and can protect cells from various external stimuli such as chemicals, osmotic stress, oxidative stress, and nutrient deficiencies (Blankenship and Mitchell, 2006). These characteristics are beneficial for the biotechnological application of biofilms as so-called biofilm reactors to produce desired compounds. Biofilm reactors are formed by the adherence of immobilized cells to solid surfaces, and they have a notable tolerance to the cytotoxic effects of substrates and products. Moreover, they improve the efficiency of the reuse of immobilized strains during batch fermentation (Pal and Khanum, 2011; Li et al., 2012). Nonetheless, biofilms are generally undesired, especially in clinical and industrial fields. Pathogenic microorganisms such as *Candida* and *Pseudomonas* species can form their biofilms on medical devices, catheters, and implants, as well as on various host tissues (Kojic and Darouiche, 2004). Biofilms formed *in vivo* by pathogenic microorganisms are difficult to remove since cells in the biofilm are highly resistant to antibiotics and host defenses (Lebeaux et al., 2014). Yeast biofilms are particularly problematic because of the strong capacity of yeast cells to adhere to abiotic surfaces and host cells. Thus, there is a need for efficient, economical, and sustainable strategies for yeast biofilm inhibition or removal.

During the first step of biofilm formation, yeast cells stick together through flocculation. This attachment happens because of specific adhesion proteins, known as GPI-anchored glycoproteins, which are encoded by genes such as *FLO1*, *FLO5*, *FLO9*, *FLO10*, and *FLO11* (Verstrepen and Klis, 2006). Among these genes, *FLO11* plays a significant role in biofilm formation, as it promotes cell-to-cell and cell-to-polystyrene adhesion, which are dependent on the presence of calcium (Váchová et al., 2011). Some studies have shown that the ability of yeast to form biofilms depends on the presence of Flo11, a protein regulated by nutritional and environmental factors (Zara et al., 2009). This regulation allows yeast to adapt to new environments, as the transition from non-biofilm to biofilm formation is necessary for survival. Expression of *FLO11* is positively regulated by the transcriptional factor Flo8 (Fidalgo et al., 2008). Although the activity of Flo8 is known to be regulated by the PKA-cAMP pathway under conditions of carbon source starvation, and by the TORC1/2 pathways under conditions of nitrogen source starvation (Halme et al., 2004), the detailed mechanisms involved in post-translational modifications of Flo8 remain unclear.

Phagocytic cells of the innate immune system, such as macrophages, recognize and engulf microorganisms such as yeast in the blood and tissues. Engulfed microorganisms are enclosed within phagosomes, which mature through a fusion process with endosomes, ultimately fusing with lysosomes to form phagolysosomes (Haas, 2007). The environment within phagolysosomes is characterized by acidity and the presence of numerous proteases and lipases, resulting in conditions of severe nutrient restriction and inhibiting microbial growth significantly. Additionally, microorganisms within phagolysosomes undergo oxidative attacks from reactive oxygen species (ROS) and reactive nitrogen species (RNS) produced by NADPH oxidase and nitric oxide synthase (NOS), respectively. The combined action of these factors allows macrophages to eliminate most engulfed microorganisms. Macrophages also induce the activation of the entire immune system by producing inflammatory cytokines such as TNF-α, IL-6, IL-8, IL-12, and IFN-γ (Netea et al., 2015). However, the emerging fungal pathogenic *Candida glabrata* has been suggested to evade the typical immune response and to survive and proliferate within macrophages (Rai et al., 2012). A comprehensive analysis using a mutant library of *C. glabrata* revealed the significant contribution of *EPA6* to biofilm formation (Iraqui et al., 2005). *EPA6* encodes an adhesin involved in biofilm formation (de Groot et al., 2013). Unlike *S. cerevisiae*, which relies on Flo11 for biofilm formation, *C. glabrata* utilizes not only Epa6 but also other GPI-anchored proteins such as Epa7 and Epa3 for substantial contributions to biofilm formation. *EPA6* and *EPA7* are expressed before and during biofilm formation, while *EPA3* is induced only during it (Iraqui et al., 2005). Therefore, the biofilm formation of *C. glabrata* is more complex than that of *S. cerevisiae*, although the details remain incompletely understood (Timmermans et al., 2018; Van Ende et al., 2019; Malinovská et al., 2023).

The present study demonstrated that Can1 plays a novel role in the control of yeast biofilm formation. The *S. cerevisiae CAN1* knockout strain exhibited enhanced Flo11-dependent aggregation, leading to increased biofilm formation. The characteristics of the *C. glabrata CAN1* knockout strain were similar to those of the *S. cerevisiae CAN1* knockout strain, with enhanced biofilm formation and retarded proliferation. Importantly, the *C. glabrata CAN1* knockout strain displayed increased pathogenicity. These findings shed light on yeast biofilm mechanisms, offering the potential for the development of antibiofilm agents in future studies.

## Materials and methods

### Culture media, strains, plasmids

Three growth media were used in this study: a synthetic complete medium (SC) (2% glucose, 0.67% yeast nitrogen base without amino acids [Difco Laboratories, Detroit, MI, United States], and 0.1% complete supplement mixture [Formedium, Norfolk, United Kingdom]) for yeast cells; a yeast extract-peptone-dextrose medium (YPD) (2% glucose, 2% peptone [Nacalai Tesque, Kyoto, Japan], and 1% yeast extract [Nacalai Tesque]) for yeast cells; and a high-glucose Dulbecco’s modified Eagle’s medium with 25 mM HEPES and 4 mM glutamine (DMEM) (FUJIFILM Wako Pure Chemical, Osaka, Japan) for mammalian cells.

Supplementary Table S1 summarizes the 11 yeast strains used in this study: 7 strains (WT, *can1*Δ, *alp1*Δ, *flo8*Δ, *seg1*Δ, *can1*Δ*flo8*Δ, and *can1*Δ*seg1*Δ) with the *S. cerevisiae* L5685 background; 2 strains (WT [BY4741 WT] and *can1*Δ [BY4741 *can1*Δ]) with the *S. cerevisiae* BY4741 background; and 2 strains (WT [CgWT] and *can1*Δ [*Cgcan1*Δ]) with the *Candida glabrata* KUE100 background. Strains *can1*Δ, BY4741 *can1*Δ, and *Cgcan1* were constructed in previous studies (Nishimura et al., 2022; Tanahashi et al., 2023; Nishimura et al., 2023b). Strain *alp1*Δ was constructed from WT by integrating the *kanMX4* gene cassette amplified by PCR with gene-specific primer sets (ALP1 deletion Fw and Rv, listed in Supplementary Table S2) and pFA6a-kanMX4 (purchased from the AddGene repository). For the construction of strains *flo8*Δ and *can1*Δ*flo8*Δ, the *FLO8* gene-specific deletion cassette containing the *hphMX6* gene was prepared by using PCR with primers (FLO8 deletion Fw and Rv, listed in Supplementary Table S2) and pFA6-hphMX6 (purchased from the AddGene repository). For the construction of strains *seg1*Δ and *can1*Δ*seg1*Δ, the *SEG1* gene-specific deletion cassette containing the *hphMX6* gene was prepared by using PCR with primers (SEG1 deletion Fw and Rv, listed in Supplementary Table S2) and pFA6-hphMX6 (purchased from the AddGene repository). The deletion cassettes were subsequently integrated into the genome in WT and *can1*Δ by transformation.

Plasmids pRS416-P_ADH1_-CAN1^WT^-yEGFP-T_CYC1_, pRS416-P_ADH1_-CAN1^G434C^-yEGFP-T_CYC1_, and pRS416-P_ADH1_-CAN1^N379I^ WT-yEGFP-T_CYC1_ were constructed previously (Tanahashi et al., 2023).

### Growth curve

Yeast cells were cultured at 30°C (for *S. cerevisiae* cells) or 37°C (for *C. glabrata* cells) in SC or DMEM medium starting from an optical density at 600 nm (OD_600_) of 0.1 under shaking conditions. Cell growth was monitored by measuring OD_600_ with a DU-800 spectrophotometer (Beckman Coulter, Brea, CA, United States).

### Isolation of the revertant from *can1Δ*

*Can1*Δ cells were passaged 5 times using glass test tubes. Each of them was cultured at 30°C in SC medium starting from an OD_600_ of 0.1 for 10–12 h. The cells were subsequently plated on SC medium. After 2 days at 30°C, 10 colonies were picked to observe their growth. The mutant whose growth rate was closest to that of WT was analyzed in this study as the revertant.

### Whole-genome sequencing

The revertant was grown in SC medium at 30°C for 2 days with shaking. After the cells were washed twice with sterile water, genomic DNA was extracted from the cells by using a Dr. GenTLE (from Yeast) High Recovery kit (Takara Bio, Shiga, Japan). Libraries for sequencing analysis were prepared using a NEB Next Ultra DNA Library Prep Kit (New England Biolabs, Ipswich, MA, United States), and paired-end short reads of 150 bp were produced using an Illumina NovaSeq 6,000 system (Illumina, San Diego, CA, United States). The sequencing processes were performed by a commercial DNA sequencing service (Rhelixa Inc., Tokyo, Japan).

### Determination of biofilm formation

Biofilm formation was evaluated by crystal violet staining as described previously (Bou Zeidan et al., 2014), with some modifications. Yeast cells were cultured to the stationary phase and resuspended at an OD_600_ of 0.3 in SC medium. The cell suspensions (3 mL) were transferred to 6-well polystyrene plates (treated with corona discharge) and incubated at 30°C (for *S. cerevisiae* cells) or 37°C (for *C. glabrata* cells) for 48 h under static conditions. After the removal of the medium, the plates were washed three times with phosphate-buffered saline (PBS). Then, 1 mL of crystal violet solution (1% in 20% ethanol) was added, and the plates were left to stand at room temperature for 30 min. After the plates were washed three times with PBS, crystal violet was extracted by the addition of 1 mL ethanol. Biofilm formation was determined by measuring the A_570_ of supernatants.

### Flocculation assay

Yeast cells were cultured to the stationary phase and resuspended in 0.9% NaCl solution with/without 10 mM CaCl_2_ at an OD_600_ of 0.6. Sedimentation was monitored at OD_600_ with the DU-800 spectrophotometer.

### Quantitative PCR analysis of *FLO11*

**Figure 1:**
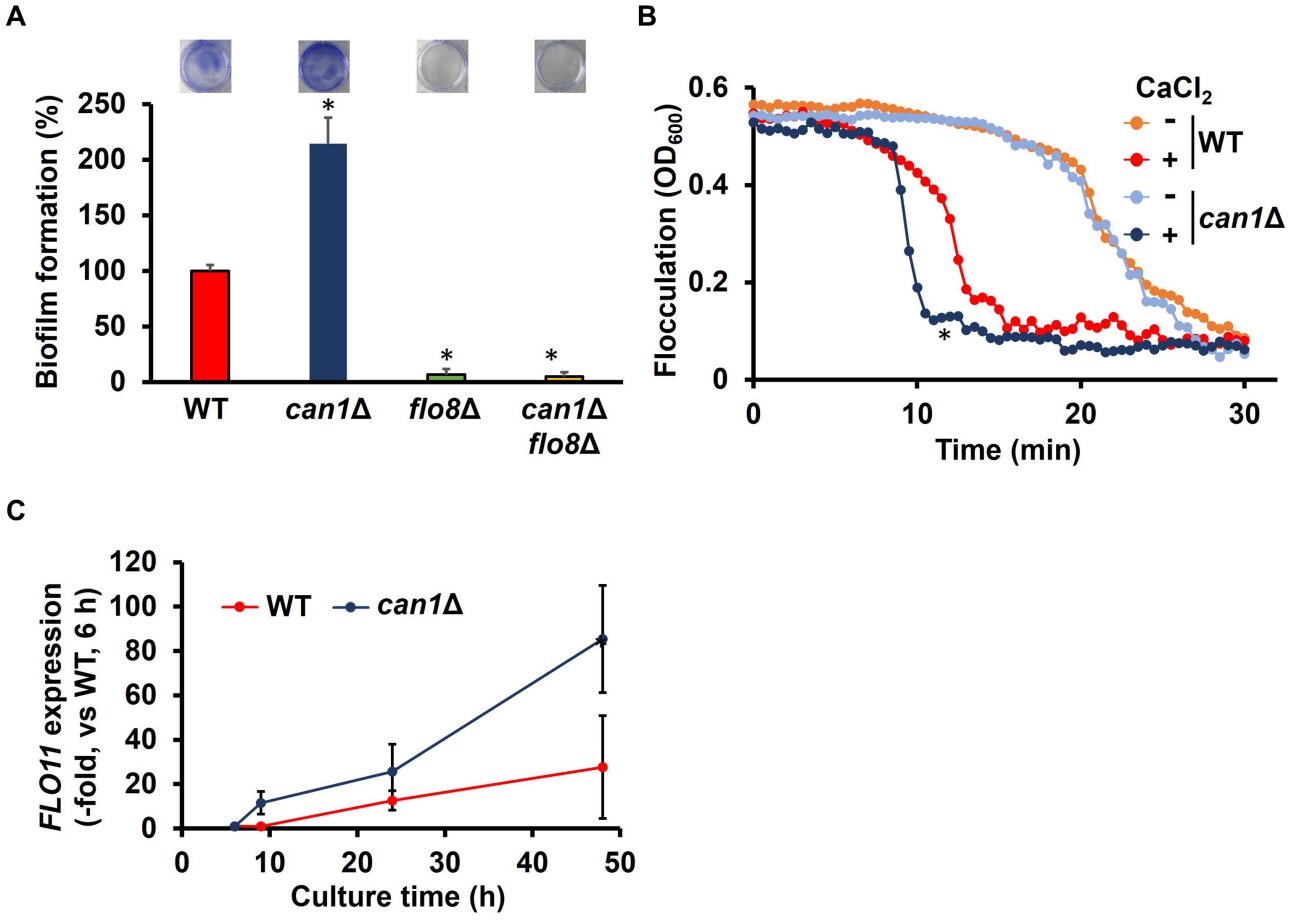
Enhanced biofilm formation in *can1*Δ. **(A)** Biofilm formation of WT, *can1*Δ, *flo8*Δ, and *can1*Δ*flo8*Δ cells. The upper photographs show biofilms stained with crystal violet, while the lower panel indicates quantitation of biofilm formation. Biofilm formation of WT was taken as 100%. Data are presented as means ± SD (*n* = 6), and statistical significance was determined by one-way ANOVA with Tukey’s test. ^*^p < 0.05 versus WT, ^**^p < 0.05 versus *can1*Δ. **(B)** Flocculation assay. Flocculation of WT and *can1*Δ cells was evaluated by measuring the OD_600_ of the cell suspensions in the presence or absence of CaCl_2_ (10 mM). Three independent experiments were performed, but representative results are presented here due to the large variation in absolute values. Statistical significance was determined by two-way ANOVA with Tukey’s test. ^*^p < 0.05 versus WT + CaCl_2_. **(C)** *FLO11* expression. The *FLO11* mRNA levels of WT and *can1*Δ were determined by qPCR under the biofilm formation condition. *FLO11* expression of WT at 6 h was assigned a value of 1.0. *ACT1* mRNAs were used in gene expression normalization. Data are presented as means ± SD (*n* = 3), and statistical significance was determined by two-way ANOVA with Tukey’s test. ^*^p < 0.05 versus WT.

Yeast cells were grown to the stationary phase and inoculated into SC medium starting from an OD_600_ of 0.3. After incubation at 30°C for the times indicated in Figure 1C under static conditions, the cells were disrupted by using a multi-beads shocker (MB601U; Yasui Kikai, Osaka, Japan) with 0.5-mm glass beads. The total RNA was extracted with a NucleoSpin RNA Plus kit (Takara Bio) according to the manufacturer’s instructions. cDNA was synthesized from the total RNA with a PrimeScript RT reagent kit (Takara Bio). The relative abundance of *FLO11* mRNAs was quantified by means of qPCR with a Light Cycler 96 system (Roche, Basel, Switzerland) and SsoAdvanced Universal SYBR Green Supermix (Bio-Rad Laboratories, Hercules, CA, United States). The following primer sets (listed in Supplementary Table S2) were used in this analysis: *FLO11* qPCR Fw and Rv (PCR efficiency: 94.1%); and *ACT1* qPCR Fw and Rv (PCR efficiency: 97.8%). The following PCR protocol was used: 95°C for 4 min followed by 40 cycles of denaturation at 95°C for 15 s and annealing/extension at 60°C for 30 s. *ACT1* mRNAs were used in gene expression normalization. The cycle threshold of each gene was normalized to that of the housekeeping gene *ACT1*, and relative expression levels were calculated using the 2^−ΔΔCt^ method.

### Oxidative stress tolerance

Yeast cells were cultured to the stationary phase and resuspended at an OD_600_ of 0.3 in SC medium. The cell suspensions (3 mL) were transferred to 6-well polystyrene plates and incubated at 37°C for 48 h under static conditions to facilitate biofilm formation. To prepare planktonic cells, the cell suspensions (5 mL) were transferred to conical polypropylene tubes and cultured at 37°C for 48 h under shaking conditions. The medium was removed, and the cells were washed three times with PBS and treated with 10 mM or 50 mM H_2_O_2_. After 1 or 2 h of treatment, the cells were plated on YPD. Cell survival rates were calculated by normalizing the number of colony-forming units (CFUs) at each time point to the CFUs before the treatment with H_2_O_2_.

### Labeling of *Candida glabrata*

Yeast cells were cultured to the stationary phase and washed with water. The cells were treated with 400 μg/mL fluorescein isothiocyanate isomer-I (FITC; Nacalai Tesque) in 100 mM bicarbonate buffer (pH 9.0) or 200 μg/mL Alexa647-conjugated Concanavalin A (ConA; Thermo Fisher Scientific, Waltham, MA, United States) in PBS for 30 min under dark conditions and were then washed three times with PBS. Labeling efficiency was calculated by using a BD Accuri C6 flow cytometer (BD Biosciences, Franklin Lakes, NJ, United States).

### Cell culture

RAW264.7 cells expressing apoptosis-associated speck-like protein containing a CARD domain (ACS) (InvivoGen, San Diego, CA, United States) were cultured in DMEM supplemented with 10% heat-inactivated FBS (MP Biomedicals, Solon, OH, United States) and 1% penicillin–streptomycin under standard cell culture conditions. When necessary, cells were stained with Plasmem Bright Green (Dojindo, Kumamoto, Japan) according to the manufacturer’s instructions.

### Parasitism proportion

RAW264.7 cells (3.6 × 10^5^ cells) were cultured on 35 mm glass-bottom dishes for 1 day and then infected with ConA-labeled *C. glabrata* cells at a multiplicity of infection (MOI) of 5. After culturing for 2 h under standard cell culture conditions, the dishes were washed 3 times with PBS. The cells were fixed with 4% paraformaldehyde for 15 min and then observed by using an SP8 FALCON confocal microscope (Leica, Wetzlar, Germany). The parasitism proportion was calculated by counting the internalized yeast cells per RAW264.7 cell.

### Proliferation of *Candida glabrata* cells in macrophages

**Figure 2:**
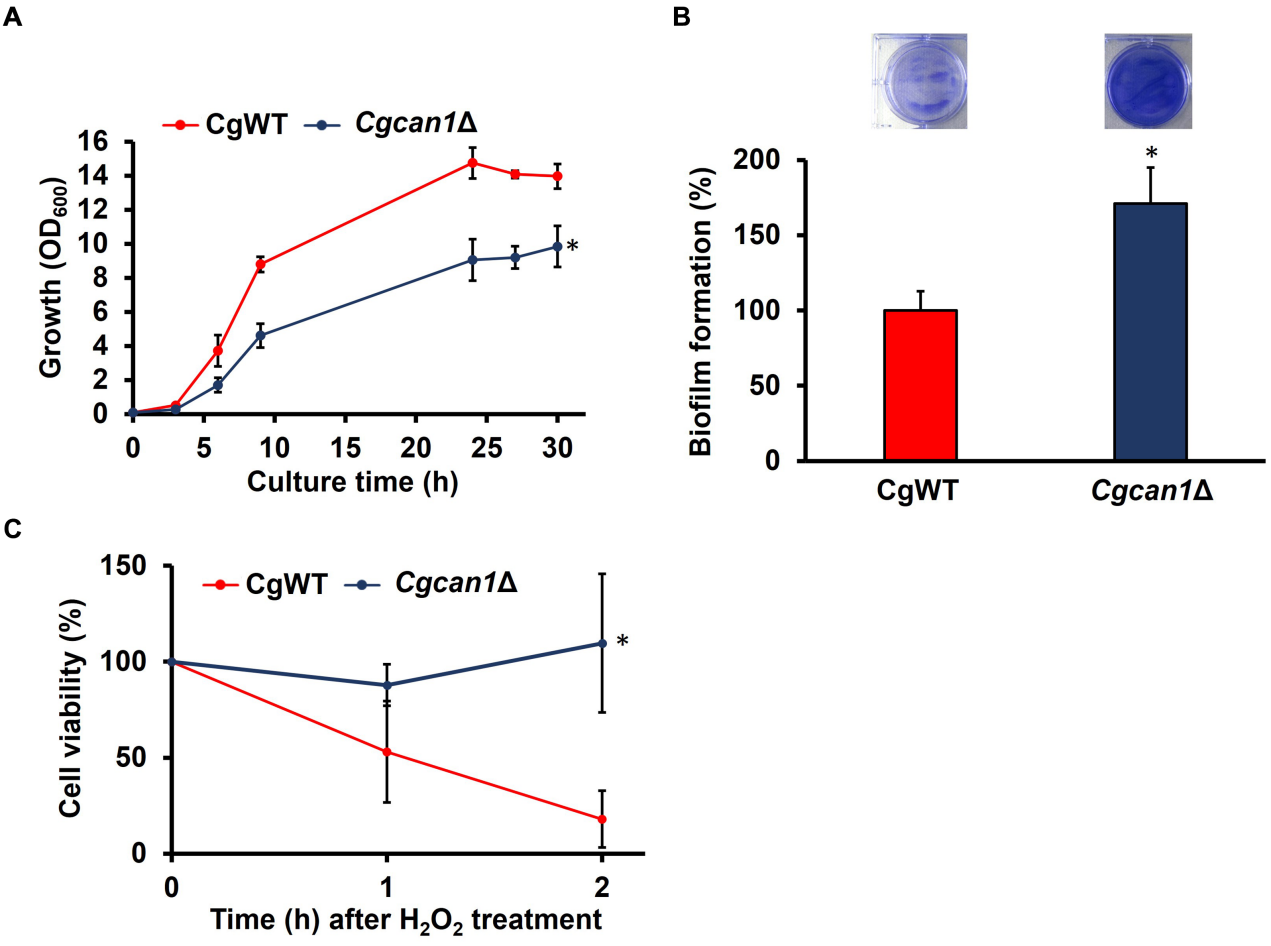
Enhanced biofilm formation in *Cgcan1*Δ. **(A)** Growth curve. Cell growth in SC medium was determined at the indicated time points by measuring OD_600_. Data are presented as means ± SD (*n* = 3), and statistical significance was determined by two-way ANOVA with Tukey’s test. ^*^p < 0.05, versus CgWT. **(B)** Biofilm formation. The upper photographs show biofilms stained with crystal violet, while the lower panel indicates the quantitation of biofilm formation. Biofilm formation of CgWT was taken as 100%. Data are presented as means ± SD (*n* = 6), and statistical significance was determined by Student’s *t*-test. ^*^p < 0.05 versus CgWT. **(C)** Oxidative stress tolerance. Cell viability was determined by measuring colony-forming units. Cells after treatment with 50 mM H_2_O_2_ were subjected to viability assessment at the indicated time points. Cell viability before H_2_O_2_ treatment was taken as 100. Data are presented as means ± SD (*n* = 3), and statistical significance was determined by two-way ANOVA with Tukey’s test. ^*^p < 0.05 versus CgWT.

RAW264.7 cells (3.6 × 10^5^ cells) were cultured on 6-well plates for 1 day and then infected with *C. glabrata* cells at an MOI of 5. After culturing for 2 h under standard cell culture conditions, the plates were washed three times with PBS. The cells were re-cultured for the times indicated in Figure 2B. The intracellular yeast cells were prepared by the disruption of RAW264.7 cells with 0.1% Triton X-100 and were plated on YPD. Viable cell numbers were determined by measuring CFUs.

### Macrophage damage assay

RAW264.7 cells (3.6 × 10^5^ cells) were cultured on 6-well plates for 1 day and then infected with *C. glabrata* cells at an MOI of 5. After culturing for 2 h under standard cell culture conditions, the plates were washed three times with PBS. The cells were re-cultured for the times indicated in Figure 2C. The release of lactate dehydrogenase (LDH) from the cells was determined by measuring LDH activity in supernatants. LDH activity was measured using an LDH cytotoxicity assay kit (Nacalai Tesque).

### Expression of biofilm-related genes of *Candida glabrata* in macrophages

RAW264.7 cells (1.5 × 10^6^ cells) were cultured on 100 mm dishes for 1 day and then infected with *C. glabrata* cells at an MOI of 5. After culturing for 2 h under standard cell culture conditions, the dishes were washed three times with PBS. The cells were re-cultured for 9 h and were disrupted by using a multi-beads shocker (MB601U; Yasui Kikai) with 0.5-mm glass beads. The total RNA was extracted with a NucleoSpin RNA Plus kit (Takara Bio) according to the manufacturer’s instructions. cDNA was synthesized from the total RNA with a PrimeScript RT reagent kit (Takara Bio). The relative abundance of *EPA1*, *EPA*2, *EPA3*, *EPA6*, and *EPA7* mRNAs was quantified by means of qPCR with a Light Cycler 96 system (Roche) and SsoAdvanced Universal SYBR Green Supermix (Bio-Rad Laboratories). The following primer sets (listed in Supplementary Table S2) were used in this analysis: *EPA1* qPCR Fw and Rv (PCR efficiency: 92.9%); *EPA2* qPCR Fw and Rv (PCR efficiency: 98.1%); *EPA3* qPCR Fw and Rv (PCR efficiency: 93.0%); *EPA6* qPCR Fw and Rv (PCR efficiency: 91.9%); *EPA7* qPCR Fw and Rv (PCR efficiency: 97.3%); and *CgACT1* qPCR Fw and Rv (PCR efficiency: 98.3%). The following PCR protocol was used: 95°C for 4 min followed by 40 cycles of denaturation at 95°C for 15 s and annealing/extension at 60°C for 30 s. The cycle threshold of each gene was normalized to that of the housekeeping gene *CgACT1*, and relative expression levels were calculated using the 2^−ΔΔCt^ method.

### Expression of cytokine genes under infection

RAW264.7 cells (3.6 × 10^5^ cells) were cultured on 6-well plates for 1 day and then infected with *C. glabrata* cells at an MOI of 5. After culturing for 2 h under standard cell culture conditions, the plates were washed three times with PBS. The cells were re-cultured for 24 or 48 h and subsequently collected by scrapers. The total RNA was extracted with a NucleoSpin RNA Plus kit (Takara Bio) according to the manufacturer’s instructions. cDNA was synthesized from the total RNA with a PrimeScript RT reagent kit (Takara Bio). The relative abundance of mRNAs of cytokine genes (*IL-1β*, *IL-6*, *TNF-α*, *IL-10*, and *GM-CSF*) was quantified by means of qPCR with a QuantStudio 3 real-time PCR system (Thermo Fisher Scientific) and SsoAdvanced Universal SYBR Green Supermix (Bio-Rad Laboratories). The following primer sets (listed in Supplementary Table S2) were used in this analysis: *IL-1β* qPCR Fw and Rv (PCR efficiency: 90.1%); *IL-6* qPCR Fw and Rv (PCR efficiency: 94.2%); *TNF-α* qPCR Fw and Rv (PCR efficiency: 98.1%); *IL-10* qPCR Fw and Rv (PCR efficiency: 95.7%); *GM-CSF* qPCR Fw and Rv (PCR efficiency: 95.3%); and *Mouse GAPDH* qPCR Fw and Rv (PCR efficiency: 96.3%). The following PCR protocol was used: 95°C for 4 min followed by 40 cycles of denaturation at 95°C for 15 s and annealing/extension at 60°C for 30 s. The cycle threshold of each gene was normalized to that of the mouse housekeeping gene GAPDH, and relative expression levels were calculated using the 2^−ΔΔCt^ method.

### Infection of *Caenorhabditis elegans* with *Candida glabrata* cells

The standard *C. elegans* strain N2 Bristol (obtained from the Caenorhabditis Genetic Centre) was maintained on nematode growth medium (NGM) plates (3 g/L NaCl, 2.5 g/L peptone, 5 μg/mL cholesterol, 1 mM CaCl_2_, 1 mM MgSO_4_, 25 mM KPO_4_, 20 g/L agar) with *Escherichia coli* OP50 as a food source at 20°C. *C*. *glabrata* strains were cultured in SC medium to the stationary phase and diluted to 1 × 10^7^/mL after the cells were labeled by ConA. Then, 100 μL of each suspension was plated onto a 60 mm dish with NGM plates. Synchronized L4-stage nematodes were cultured with NGM plates with 400 μM 5-fluorodeoxyuridine for 1 day and then transferred to plates with *C. glabrata* for infection. After incubation for 3 h, 40 nematodes were transferred to fresh NGM plates. Nematodes were monitored daily to assess viability by observing mobility/immobility upon mechanical stimulation using sterile inoculating loops.

### Statistical analysis

Data are presented as means ± standard deviation (SD), and statistical significance was evaluated using Student’s *t*-test, one-way/two-way analysis of variance (ANOVA) with Tukey’s test, or the log-rank test. These analyses were performed using Prism 7 (GraphPad Software, San Diego, CA, United States). Values of *p* < 0.05 were considered statistically significant.

## Results

### Can1 is a negative regulator of biofilm formation

**Figure 3:**
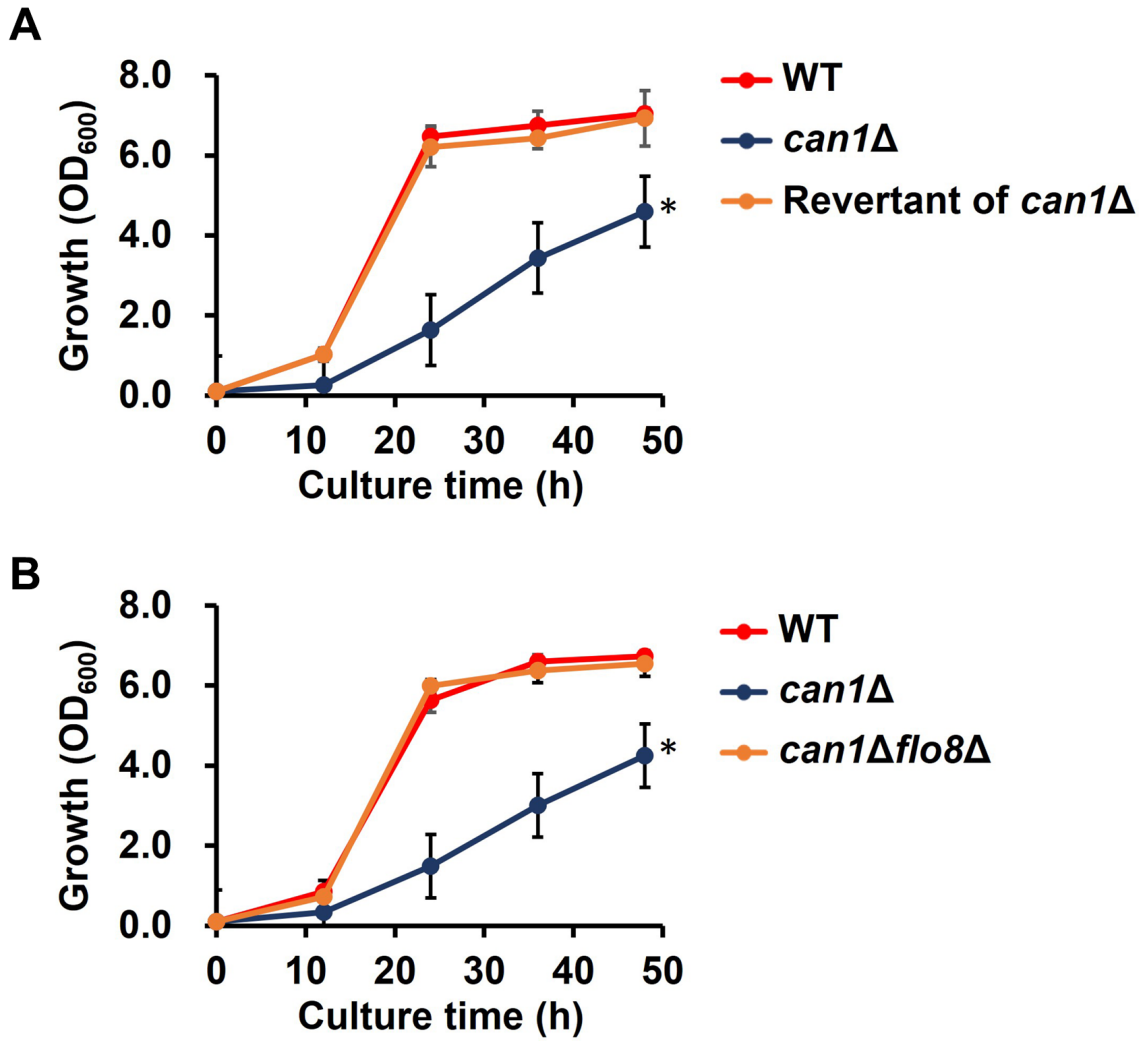
Growth defect in *can1*Δ. **(A)** Growth curve of the revertant (candidate 4) from *can1*Δ cells. Cell growth in SC medium was determined at the indicated time points by measuring OD_600_. Data are presented as means ± SD (*n* = 3), and statistical significance was determined by two-way ANOVA with Tukey’s test. ^*^p < 0.05 versus WT. **(B)** Growth curve of *can1*Δ*flo8*Δ cells. Cell growth in SC medium was determined at the indicated time points by measuring OD_600_. Data are presented as means ± SD (*n* = 3), and statistical significance was determined by two-way ANOVA with Tukey’s test. *p < 0.05 versus WT.

We first checked phenotypes of the *CAN1* gene deletion strain (*can1*Δ) established from the Σ1278b background strain. Intriguingly, *can1*Δ exhibited a significant reduction in growth rate compared to the wild-type strain (WT; Figure 3A). To elucidate the cause of the slow growth in *can1*Δ, we attempted to obtain revertant mutants with growth rates restored to the level of WT by serial passages of *can1*Δ cells in glass test tubes (Supplementary Figure S1). Figure 3A shows that the growth of candidate 4 was almost completely restored to that of WT. Whole genome sequencing analysis revealed that the revertant (candidate 4) had a deletion of a nucleotide C at position 381 on the locus of *FLO8*. This deletion led to the emergence of a stop codon at position 130 on Flo8, indicating that the revertant carries a nonsense mutation in *FLO8* (Supplementary Figure S2). Mutations with an amino acid substitution were found only in the *FLO8* locus. Next, we deleted the *FLO8* gene of *can1*Δ (*can1*Δ*flo8*Δ). As shown in Figure 3B, the growth of *can1*Δ*flo8*Δ was the same as that of WT, indicating that the disruptant of *FLO8* completely restored the growth delay observed in *can1*Δ. Previous studies reported that a nonsense mutation occurs on the *FLO8* gene of S288C background strains (Supplementary Figure S2; Liu et al., 1996). We observed the growth of *can1*Δ (BY4741 *can1*Δ) in an S288C background strain (Supplementary Figure S3). The BY4741 *can1*Δ strain displayed growth comparable to that of the WT, unlike the Σ1278b background strain, as shown in previous data. These findings suggest a genetic interaction between *CAN1* and *FLO8*.

Flo8 is the critical transcription factor for the expression of *FLO11*, which is well known for its involvement in flocculation and biofilm formation (Fidalgo et al., 2008). Considering the potential involvement of Can1 in flocculation and biofilm formation, we investigated the flocculation and biofilm formation of *can1*Δ cells. Surprisingly, *can1*Δ showed significantly greater biofilm formation than WT (Figure 1A). *FLO8* deletion abolished the enhanced biofilm formation observed in *can1*Δ (Figure 1A). *S. cerevisiae* biofilm formation initiates with calcium-dependent cell aggregation mediated by Flo11. We therefore tested the flocculation of *can1*Δ cells (Figure 1B). Under conditions without the addition of calcium ions, there was no significant difference in sedimentation rate between WT and *can1*Δ. Under calcium ion-supplemented conditions, *can1*Δ cells sedimented in around 10 min, whereas WT cells sedimented in approximately 13 min, suggesting that *CAN1* deletion enhanced Flo11-dependent flocculation. Next, the expression levels of *FLO11* under biofilm-forming conditions were measured by quantitative PCR (Figure 1C). At 6 h after the start of cultivation, there was no significant difference in the expression levels of *FLO11* between WT and *can1*Δ. However, from 6 h onwards, the expression levels of *FLO11* in *can1*Δ became much higher than those in WT, with a difference of over 3-fold observed at 48 h mark. Thus, Can1 negatively regulated Flo11-dependent flocculation, thereby controlling biofilm formation.

**Figure 4:**
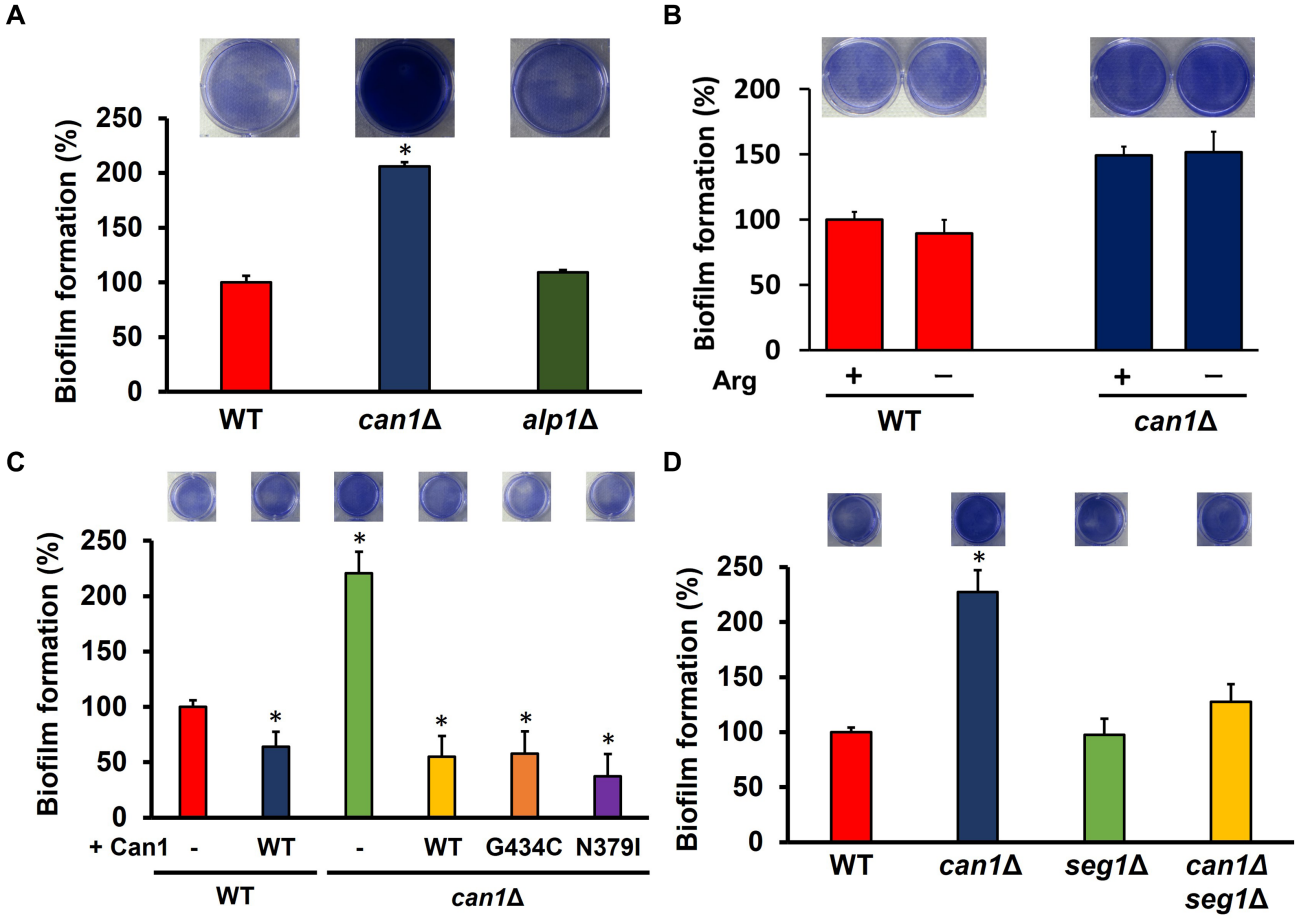
MCC/eisosome structure-dependent enhancement of biofilm formation. **(A)** Biofilm formation of WT, *can1*Δ, and *alp1*Δ cells. The upper photographs show biofilms stained with crystal violet, while the lower panel indicates the quantitation of biofilm formation. Biofilm formation of WT was taken as 100%. Data are presented as means ± SD (*n* = 6), and statistical significance was determined by one-way ANOVA with Tukey’s test. ^*^p < 0.05 versus WT. **(B)** Biofilm formation under the conditions without arginine. The upper photographs show biofilms stained with crystal violet, while the lower panel indicates the quantitation of biofilm formation. Biofilm formation of WT under the condition containing arginine was taken as 100%. Data are presented as means ± SD (*n* = 3), and statistical significance was determined by Student’s *t*-test. **(C)** Biofilm formation of *can1*Δ expressing Can1 variants. The upper photographs show biofilms stained with crystal violet, while the lower panel indicates the quantitation of biofilm formation. Biofilm formation of WT was taken as 100%. Data are presented as means ± SD (*n* = 6), and statistical significance was determined by one-way ANOVA with Tukey’s test. ^*^p < 0.05 versus + Vector. **(D)** Biofilm formation of WT, *can1*Δ, *seg1*Δ, and *alp1*Δ cells. The upper photographs show biofilms stained with crystal violet, while the lower panel indicates the quantitation of biofilm formation. Biofilm formation of WT was taken as 100%. Data are presented as means ± SD (*n* = 6), and statistical significance was determined by one-way ANOVA with Tukey’s test. ^*^p < 0.05 versus WT.

Can1 functions as a transceptor for sensing extracellular basic amino acids in addition to arginine uptake. Can1 within the MCC/eisosome may also sense changes in the external environment. We then investigated what functions of Can1 regulate biofilm formation. The biofilm-forming ability of the disrupted strain (*alp1*Δ) of *ALP1*, the paralog gene of *CAN1*, was comparable to that of WT (Figure 4A). To examine the relationship between arginine transport and biofilm-forming ability, biofilm formation was determined under arginine-free conditions (Figure 4B). The presence of arginine had little effect on biofilm formation in WT and *can1*Δ. Next, we used Can1 variants (G434C, N379I) that firmly retain arginine transporter activity but not receptor capacity for basic amino acids. The expression of Can1 WT caused a dramatic decrease in the biofilm formation of *can1*Δ (Figure 4C). Can1 G434C or N379I-expressing *can1*Δ cells exhibited the same levels of biofilm formation as WT-expressing *can1*Δ cells, indicating that the transceptor function of Can1 may not be involved in the regulation of biofilm formation (Figure 4C). Furthermore, we constructed an *SEG1* deletion strain (*can1*Δ*seg1*Δ) of *can1*Δ. Seg1 is a protein required for proper MCC/eisosome assembly; *SEG1* deletion eliminates MCC/eisosome. Figure 4D shows that *SEG1* deletion canceled the enhanced biofilm formation of *can1*Δ. These results suggested that Can1 within MCC/eisosome regulates biofilm formation.

### Can1 affects cytotoxicity and virulence

*C. glabrata* has emerged as the second most common agent of mucosal and invasive candidiasis (Roetzer et al., 2011a; Hassan et al., 2021). Understanding the mechanisms involved in stress tolerance and infection of *C. glabrata* is essential for improving clinical treatments. In our previous study, we genetically and biochemically identified the *C. glabrata* orthologue (*CgCAN1*) of the *S. cerevisiae CAN1* (Nishimura et al., 2023b). Here, we investigated whether CgCan1 regulates biofilm formation similarly to *S. cerevisiae*. The growth of a disruption strain (*Cgcan1*Δ) of the *CgCAN1* gene was first observed (Figure 2A). The data showed that the growth of *Cgcan1*Δ was remarkably slower than that of the parent strain (CgWT). Furthermore, the biofilm formation of *Cgcan1*Δ was significantly increased compared to CgWT (Figure 2B). These results indicate that CgCan1 has functions equivalent to those of the *S. cerevisiae* Can1, particularly with respect to the regulation of biofilm formation.

To determine the oxidative stress tolerance of CgWT and *Cgcan1*Δ, the number of viable cells within biofilms after treatment with hydrogen peroxide was measured by counting CFUs (Figure 2C). As the figure shows, the number of viable cells in CgWT decreased by approximately 50% compared to the untreated condition at 1 h after treatment with 50 mM hydrogen peroxide. After 2 h, the number of viable cells in CgWT further decreased by about 20%. In contrast, in *Cgcan1*Δ, cell viability did not decrease at either 1 h or 2 h after hydrogen peroxide treatment. Treatment with 10 mM hydrogen peroxide did not result in any decrease in cell viability for either strain. There was no difference in both strains in planktonic growth (Supplementary Figure S4). Thus, the enhanced biofilm-forming ability of *Cgcan1*Δ contributes to an improved tolerance to oxidative stress.

**Figure 5:**
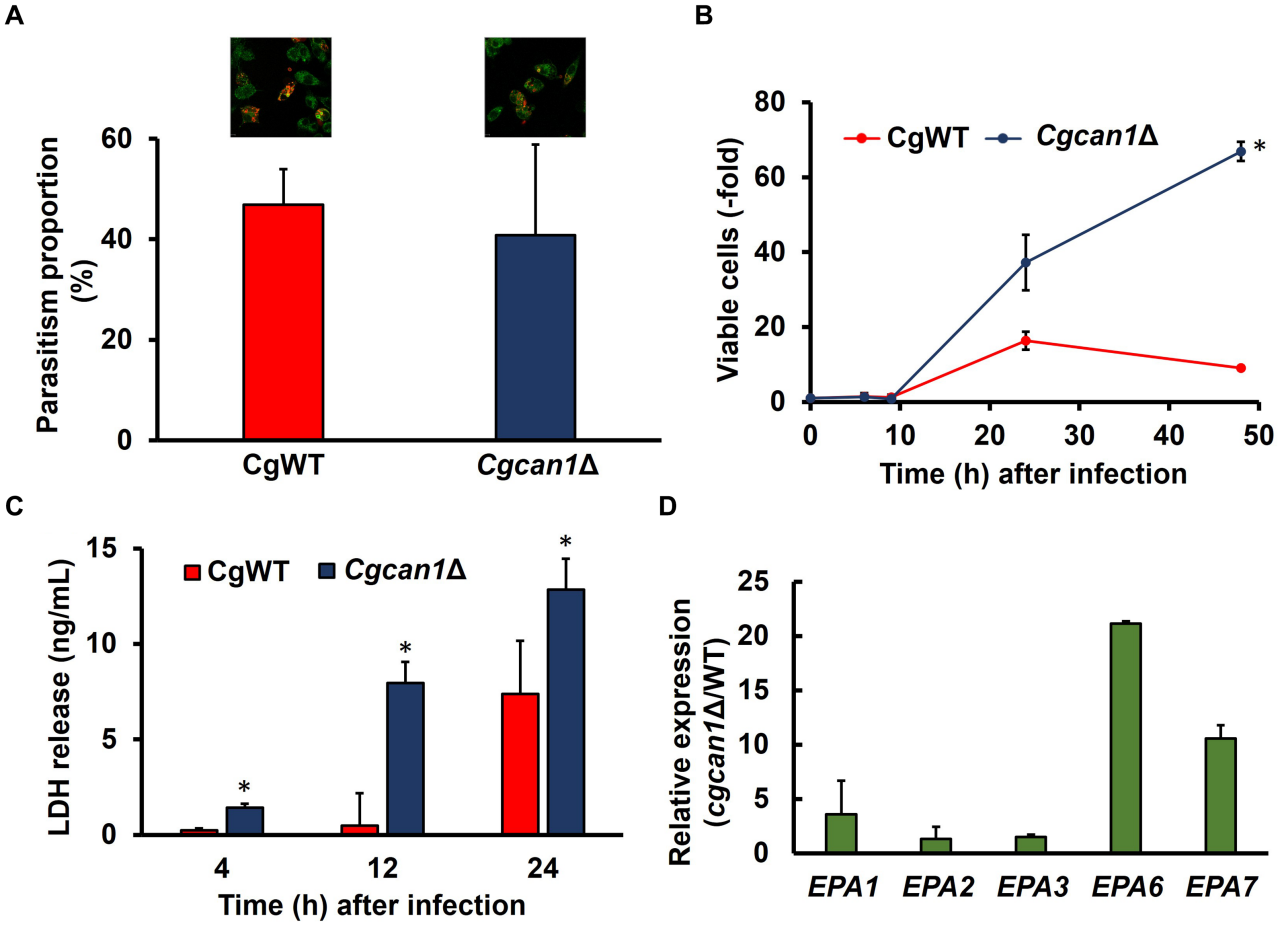
Macrophage damage by *Cgcan1*Δ. **(A)** Microscopic quantification of CgWT and *Cgcan1* phagocytosed by macrophage-like cells. Alexa647-conjugated Con A-labeled CgWT or *Cgcan1*Δ cells were mixed with ASC-expressing RAW264.7 cells stained with Plasmem Bright Green for 2 h. Green and red show RAW264.7 and *C. glabrata*, respectively. The parasitism proportion was calculated by counting the number of internalized yeast cells per macrophage cell (*n* = 100). Upper photographs show microscopic data, while the lower panel indicates the quantitation of parasitism. Data are presented as means ± SD (*n* = 3), and statistical significance was determined by Student’s *t*-test. **(B)** Proliferation of *Cgcan1*Δ in macrophage-like cells. The cellular proliferation of CgWT and *Cgcan1*Δ in ASC-expressing RAW264.7 cells was measured by counting the colony-forming units at the indicated time points after infection. The number of colonies immediately after infection was calculated as 1. Data are presented as means ± SD (*n* = 6), and statistical significance was determined by two-way ANOVA with Tukey’s test. ^*^p < 0.05 versus CgWT. **(C)** LDH release from *Cgcan1*Δ-infected macrophage-like cells. The release of LDH into the supernatant was measured as a marker for host cell damage. Data are presented as means ± SD (n = 3), and statistical significance was determined by Student’s *t*-test. **(D)** Differences in expression levels of biofilm-related genes between CgWT and *Cgcan1*Δ. Biofilm formation-related genes (*EPA1*, *EPA*2, *EPA3*, *EPA6*, and *EPA7*) were determined by qPCR at 9 h after infection. Data are shown for each mRNA level of *Cgcan1*Δ relative to CgWT (*n* = 3).

We then analyzed the relationship between biofilm formation and toxicity in *C. glabrata*. Initially, we examined growth in DMEM (a standard animal cell culture medium). Supplementary Figure S5 reveals that the proliferation of *Cgcan1*Δ was markedly slow in DMEM medium, just as it was in SC medium. To determine the initial intracellular parasitism, *C. glabrata* cells were fluorescently labeled for the microscopic detection of cells. As shown in Supplementary Figure S6, the labeling efficiency of FITC was approximately 10%, while that of Alexa Fluor 647-conjugated Concanavalin A was approximately 100%. Therefore, Concanavalin A was suitable as a labeling reagent for *C. glabrata*. Next, we investigated the initial intracellular parasitism of CgWT and *Cgcan1*Δ in mouse macrophage-like cells (RAW264.7 cells expressing ASC). There was no significant difference between two strains in regard to intracellular parasitism at the early stage (Figure 5A). We then measured the number of viable cells inside RAW264.7 cells for CgWT and *Cgcan1*Δ by counting CFUs (Figure 5B). Up to 9 h post-infection, there was no significant difference in the number of viable cells between CgWT and *Cgcan1*Δ. However, after 24 h, the number of viable cells in *Cgcan1*Δ was significantly higher than in CgWT, and a 7-fold difference was observed after 48 h. Furthermore, we determined the cytotoxicity of CgWT and *Cgcan1*Δ against RAW264.7 cells based on LDH activity in the culture supernatant (Figure 5C). LDH is a highly stable enzyme in the cytoplasm, and its release into the culture medium indicates cell membrane damage or injury. At 4 h post-infection, there was no significant difference in LDH activity in the culture supernatant infected with either CgWT or *Cgcan1*Δ. However, at 12 h post-infection, while there was no change in LDH activity in cells infected with CgWT, a significant increase was observed in cells infected with *Cgcan1*Δ. Additionally, at 24 h, LDH activity increased in CgWT, but in *Cgcan1*Δ, a 2-fold increase in LDH activity was observed. To examine biofilm formation by *C. glabrata* within macrophage-like cells, we analyzed the expression of biofilm-related genes (*EPA1*, *EPA2*, *EPA3*, *EPA6*, *EPA7*) under infection conditions (Figure 5D). The results showed that the expression of biofilm-related genes was higher in *Cgcan1*Δ than in CgWT, and in particular, the expression of *EPA6* and *EPA7* was significantly increased.

**Figure 6:**
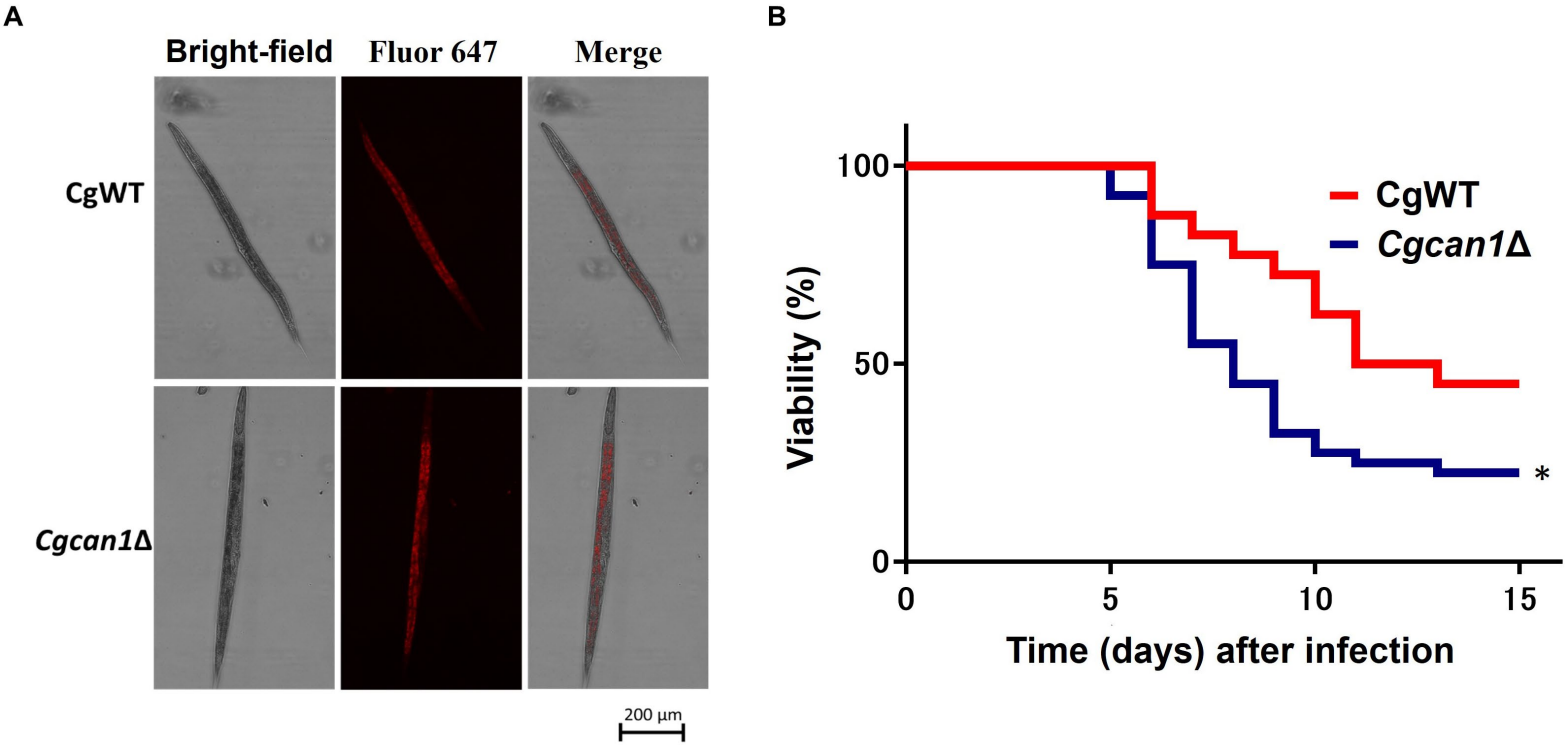
Survival curves of *Candida elegans* infected with *Cgcan1*Δ. **(A)** Feeding of *C. glabrata* by *C. elegans*. Alexa647-conjugated Con A-labeled CgWT and *Cgcan1*Δ were fed to *C. elegans*. **(B)** Survival curves. Survival numbers of *C. elegans* infected with CgWT or *Cgcan1*Δ are shown at the indicated days. Data are presented as means ± SD (*n* = 40), and the log-rank test was used to determine statistical significance. ^*^p < 0.05 versus CgWT.

Next, we considered the potential impact on the immune function of mouse macrophage-like cells. Therefore, we examined the expression of cytokine genes (IL-1β, IL-6, IL-10, TNF-α, and GM-CSF) after infection (Supplementary Figure S7). However, at least for the cytokine genes examined in this study, there was no significant difference in expression between CgWT and *Cgcan1*Δ during infection. Finally, we investigated the pathogenicity (toxicity) of *Cgcan1*Δ at the *in vivo* level using the nematode *C. elegans*, a gut infection model. First, we examined whether *C. elegans* feeds on *C. glabrata* cells. Figure 6A shows that the nematodes fed on CgWT and Cgcan1Δ to the same degree. More importantly, *C. elegans* individuals fed with *Cgcan1*Δ exhibited a significantly reduced survival rate compared to those fed with CgWT (Figure 6B). Thus, the data clearly showed that *Cgcan1*Δ is more toxic than CgWT.

## Discussion

Yeasts such as *S. cerevisiae* survive in diverse environments by effectively sensing and adapting to external conditions. MCC/eisosome may be a macro-structure that plays an important role in environmental responses. It may sense changes in cell membrane composition and fluidity with environmental changes, prompting the transmission of signals downstream (Foderaro et al., 2017). In particular, when carbon or nitrogen sources are starved, the synthesis pathways of ergosterol and phospholipids slow down, leading to changes in cell membrane composition, which in turn leads to MCC/eisosome structural changes (Fröhlich et al., 2014). MCC/eisosome may associate with TORC2, a protein kinase complex similar to TORC1, and is localized in close proximity to TORC2 on the cell membrane. Proteins such as Slm1/Slm2 and Avo2 have been observed to shuttle between these complexes (Babst, 2019). Moreover, a recent study showed that Pil1 and tetraspan membrane proteins (Sur7, Fmp45, Pun1, Ynl194c, Nce102, and Fhn1) within MCC/eisosome may cooperatively regulate the TORC2-Ypk1 signaling (Sakata et al., 2022). While direct evidence of the MCC/eisosome–TORC2 relationship has not yet been found, the existence of a mechanism for signal transduction between MCC/eisosome and TORC2 is plausible. TORC2 modulates endocytosis based on growth conditions, suggesting the existence of an MCC/eisosome-TORC2 pathway involved in sensing environmental cues and triggering endocytosis. Recent studies have demonstrated that carbon and nitrogen source depletion induces Can1 endocytosis, indicating that Can1 plays a role as an environmental sensor (Laidlaw et al., 2021). Intriguingly, a previous investigation suggested that Can1 endocytosis regulates (and potentially inhibits) biofilm formation (Nishimura et al., 2022). Considering these insights, it is hypothesized that under nutrient depletion, structural changes in MCC/eisosome lead to Can1 endocytosis, thereby controlling biofilm formation, possibly through the activation of TORC2. Future investigations will elucidate the mechanism underlying biofilm control involving Can1 and TORC2.

The correlation between the biofilm-forming capacity of pathogenic fungi and toxicity has been extensively investigated. However, many of these studies primarily focused on analysis of strains exhibiting deficient (or significantly diminished) biofilm-forming abilities, rendering them susceptible to elimination during the early stages of host immune responses. This study presents a unique perspective by investigating a rare mutant strain (*Cgcan1*Δ) characterized by enhanced biofilm-forming capabilities, offering invaluable insights into biofilm formation dynamics and toxicity mechanisms. *CAN1* was initially identified in *S. cerevisiae* as a resistance determinant against canavanine, a toxic arginine analog (Larimer et al., 1978). Intriguingly, within the pathogenic fungus *Cryptococcus* sp., strains exhibiting high pathogenicity have been historically screened using media supplemented with canavanine (Klein et al., 2009). Although the precise relationship between canavanine resistance and pathogenicity remains elusive, the empirical screening approach may show a potential link between *CAN1* deficiency-induced enhancement of biofilm formation and pathogenicity. Future investigations should focus on canavanine-resistant *Cryptococcus* strains from a biofilm-centric perspective. Comparative analyses of *CAN1* sequences among strains exhibiting varying degrees of pathogenicity could be conducted to elucidate predictive markers for pathogenicity based on Can1 structural characteristics.

*C. glabrata* can be engulfed by macrophages but can survive and proliferate within phagosomes (Rai et al., 2012). Since the nutrient-poor environment within the phagosome induces *C. glabrata* biofilm formation, it is plausible that biofilm formation may help cells survive and proliferate within phagosomes. This study indicates that *C. glabrata* biofilm is less likely to trigger a host cytokine storm (excessive cytokine production) that leads to cellular damage. Biofilm formation is well known to confer resistance to various stresses (Seneviratne et al., 2008), consistent with the results of the hydrogen peroxide sensitivity test (50 mM) in this study. However, oxidative stress-sensitive strains of *C. glabrata* showed macrophage infection ability similar to that of CgWT, which was tolerant to 10 mM hydrogen peroxide. ROS produced by macrophages during *C. glabrata* infection are in the millimolar (mM) range (Roetzer et al., 2011b). Therefore, the high oxidative stress tolerance of *Cgcan1*Δ is less likely to contribute to higher toxicity. Some reports have suggested that infective fungi increase their volume, breaking the phagosome (Seider et al., 2011). In other words, the phagosome can hold a surprisingly small volume, and its volume limit is a potential factor in its rupture. Biofilms contain not only fungal cells but also large amounts of extracellular matrix (polysaccharides, proteins, nucleic acids, etc.) that can contribute to volume. Therefore, *Cgcan1*Δ may proliferate slowly but can surpass the phagosome’s volume tolerance faster than CgWT due to the increased volume through biofilm formation. Thus, the strong toxicity of *Cgcan1*Δ may be due to that biofilm-increased volume.

In this study, we found that the arginine transporter Can1 plays a novel role in biofilm formation control in two yeast species, *S. cerevisiae* and *C. glabrata*. Moreover, our data demonstrated that *CgCAN1* deletion enhances pathogenicity in *C. glabrata*. The present findings could contribute to our understanding of fungal biofilm-related infections and provide potential targets for antifungal therapies.

## Data availability statement

Whole genome sequence data supporting the findings of this study are available in the DDBJ Sequenced Read Archive under accession number DRR352982.

## Ethics statement

Ethical approval was not required for the studies on animals in accordance with the local legislation and institutional requirements because only commercially available established cell lines were used.

## Author contributions

AN: Conceptualization, Data curation, Formal analysis, Funding acquisition, Investigation, Methodology, Project administration, Resources, Software, Supervision, Validation, Visualization, Writing – original draft, Writing – review & editing. RT: Data curation, Writing – original draft. KN: Data curation, Writing – original draft. YM: Data curation, Writing – original draft. HT: Conceptualization, Writing – review & editing.
