# Supplementary material for: The arginine transporter Can1 negatively regulates biofilm formation in yeasts
Source: Front Microbiol. 2024 Jun 5;15:1419530. doi: 10.3389/fmicb.2024.1419530 (PMC11188447; doi:10.3389/fmicb.2024.1419530)
Supplement: Supplementary file 1 [file Data_Sheet_1.PDF]

Supplementary Material

The Arginine Transporter Can1 Negatively Regulates Biofilm Formation in Yeasts.

Akira Nishimura[1*], Ryoya Tanahashi[2,3], Kazuki Nakagami[1], Yuto Morioka[1], and Hiroshi Takagi[1]

[1]Division of Biological Science, Graduate School of Science and Technology, Nara Institute of Science and Technology, 8916-5 Takayama-cho, Ikoma, Nara 630-0192, Japan
[2]Division for Research Strategy, Institute for Research Initiatives, Nara Institute of Science and Technology, 8916-5 Takayama-cho, Ikoma, Nara 630-0192, Japan
[3]Department of Food Science and Technology, University of California Davis, One Shields Ave, Davis, CA, 95616, USA

***Correspondence**
Akira Nishimura: nishimura@bs.naist.jp; Hiroshi Takagi: hiro@bs.naist.jp

This PDF file includes:
Supplementary Tables 1-2
Supplementary Figures 1-7

Supplementary Table 1. Yeasts used in this study.

Abbreviation	Genotype	Description
WT	*MAT*a *ura3-52 trp1*	*S. cerevisiae* L5685 strain, supplied by Dr. Gerald R. Fink (Whitehead Institute)
*can1*Δ	*MAT*a *ura3-52 trp1 can1*::*kanMX4*	*CAN1*-disrupted strain with an L5685 background
*alp1*Δ	*MAT*a *ura3-52 trp1 alp1*::*kanMX4*	*ALP1*-disrupted strain with the L5685 background
*flo8*Δ	*MAT*a *ura3-52 trp1 flo8*::*hphMX6*	*FLO8*-disrupted strain with the L5685 background
*seg1*Δ	*MAT*a *ura3-52 trp1 seg1*::*hphMX6*	*SEG1*-disrupted strain with the L5685 background
*can1*Δ*flo8*Δ	*MAT*a *ura3-52 trp1 can1*::*kanMX4 flo8*::*hphMX6*	*CAN1 and FLO8*-disrupted strain with the L5685 background
*can1*Δ*seg1*Δ	*MAT*a *ura3-52 trp1 can1*::*kanMX4 seg1*::*hphMX6*	*CAN1 and SEG1*-disrupted strain with the L5685 background
BY4741 WT	*MAT*a *his3Δ1 leu2Δ0 met15Δ0 ura3Δ0*	*S. cerevisiae* BY4741 strain, obtained from Euroscarf
BY474 *can1*Δ	*MAT*a *his3Δ1 leu2Δ0 met15Δ0 ura3Δ0, can1*::*hphMX6*	*CAN1*-disrupted strain with the BY4741 background
CgWT	*his3*	*C. glabrata* KUE100 strain, supplied by Dr. Hiroji Chibana (Chiba University)
*Cgcan1*Δ	*his3 can1*::*kanMX4*	*CAN1*-disrupted strain with the KUE100 background

Supplementary Table 2. **Oligonucleotides used in this study.**

Name	Sequence (5' → 3')
ALP1 deletion Fw	CTCTTCTGTAGCACAATAGACATATATTAGCGGCAAAATTGTAGTGTTGCGATTATTGCCCGTACGCTGCAGGTCGAC
ALP1 deletion Rv	ACATACATACATATATATATATATATATATATGTGTGTGTGGTATGGAGTATTATTCTAAAAATCGATGAATTCGAGCTCG
FLO8 deletion Fw	AAAAATAAACACGAAGACGTTTATAGACATAAATAAAGAGGAAACGCATTCCGTGGTAGACGTACGCTGCAGGTCGAC
FLO8 deletion Rv	TTATTATGTTTCCTGTCATTAAGAGTTTTTATTTTTTATTATAATACTCAACACGTGACTATCGATGAATTCGAGCTCG
SEG1 deletion Fw	ACCAATTCCACAAATCACCAGCCGTTCGTTCCTAAAAATGCGGATCCCCGGGTTAATTAA
SEG1 deletion Rv	ATAACTTGTACAAGGAGAGAATTTCTGCCGAGAGTCCCTAGAATTCGAGCTCGTTTAAAC
FLO11 qPCR Fw	AGGTTCAAATGGTGCCAAGA
FLO11 qPCR Rv	AGCCACGCTAGAAGCAGAAG
ACT1 qPCR Fw	CACCAACTGGGACGATATGGA
ACT1 qPCR Rv	GGCAACTCTCAATTCGTTGTAGAA
EPA1 qPCR Fw	ACAGCGAGGAACACAATAGCA
EPA1 qPCR Rv	AGCAAAAGTTGAGTGTATCCCA
EPA2 qPCR Fw	GGCAACAACGGCAATGGTAA
EPA2 qPCR Rv	GCAGCCCTAAATCCTTCACCT
EPA3 qPCR Fw	TGGATGTTCTCCTCAGGATGTTG

EPA3 qPCR Rv	TGTAGACCAGTTGTTTGAGCCTTG
EPA6 qPCR Fw	TGATTATTTGAAATCAGGATCGAATC
EPA6 qPCR Rv	TGTCATTGTCAATGGTGTACGATAG
EPA7 qPCR Fw	GATTTACGGAAGAATGGTTCGTAC
EPA7 qPCR Rv	GGTAAATGATCTATTTCGGGTGTG
CgACT1 qPCR Fw	CTGTCTGGATCGGTGGTTCT
CgACT1 qPCR Rv	GATGGACCACTTTCGTCGTA
IL-1β qPCR Fw	GACCTTCCAGGATGAGGACA
IL-1β qPCR Rv	AGCTCATATGGGTCCGACAG
IL-6 qPCR Fw	TCCAGTTGCCTTCTTGGGAC
IL-6 qPCR Rv	GTGTAATTAAGCCTCCGACTTG
TNF-α qPCR Fw	GAGCTGTGGGGAGAACAAAAGGA
TNF-α qPCR Rv	TTGGCCCTTGAAGAGGACCTG
IL-10 qPCR Fw	GCTCTTACTGACTGGCATGAG
IL-10 qPCR Rv	CGCAGCTCTAGGAGCATGTG
GM-CSF qPCR Fw	AACCTCCTGGATGACATGCCTG
GM-CSF qPCR Rv	AAATTGCCCCGTAGACCCTGCT
Mouse GAPDH Fw	AGAAGGCTGGGGCTCATTTG
Mouse GAPDH Rv	AGGGGCCATCCACAGTCTTC

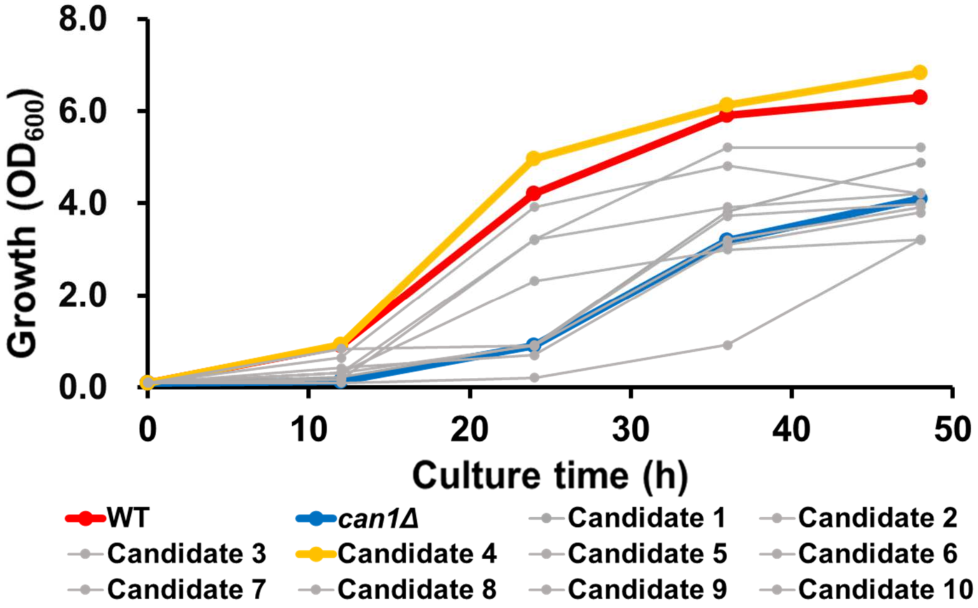

Supplementary Figure 1. Growth curve of revertant mutants from *can1*Δ. Cell growth in the SC medium was determined at the indicated time points by measuring OD_{600}. Data are presented as means ± SD (n=2).

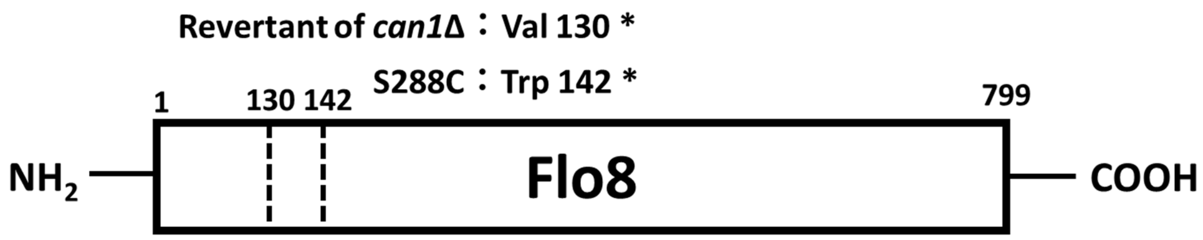

Supplementary Figure 2. Amino acid substitution in Flo8 observed in the revertant mutant (candidate 4). The revertant mutant has a stop codon at position 130, while a strain with the S288C background has a stop codon at position 142.

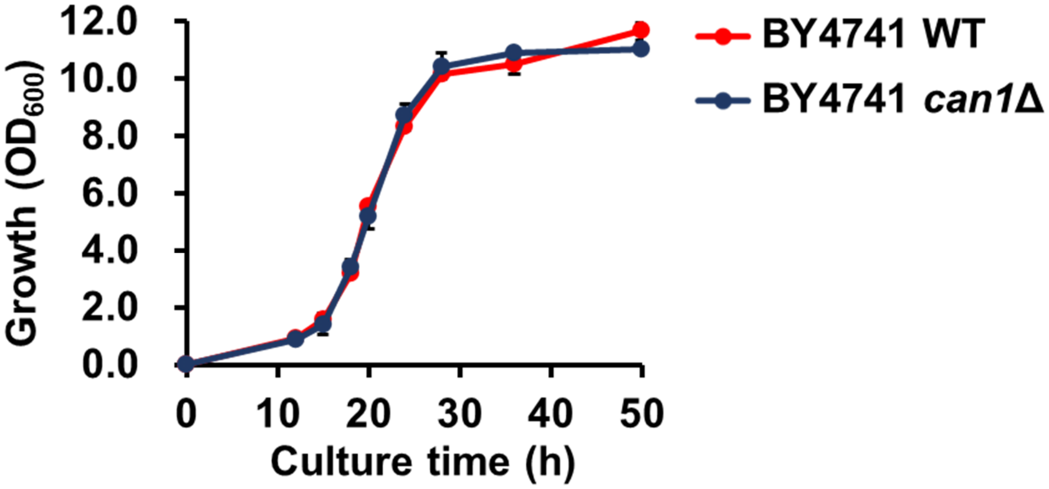

Supplementary Figure 3. Growth curve of BY4741 *can1Δ*. Cell growth in the SC medium was determined at the indicated time points by measuring OD_{600}. Data are presented as means ± SD (n=3), and statistical significance was determined by two-way ANOVA with Tukey's test.

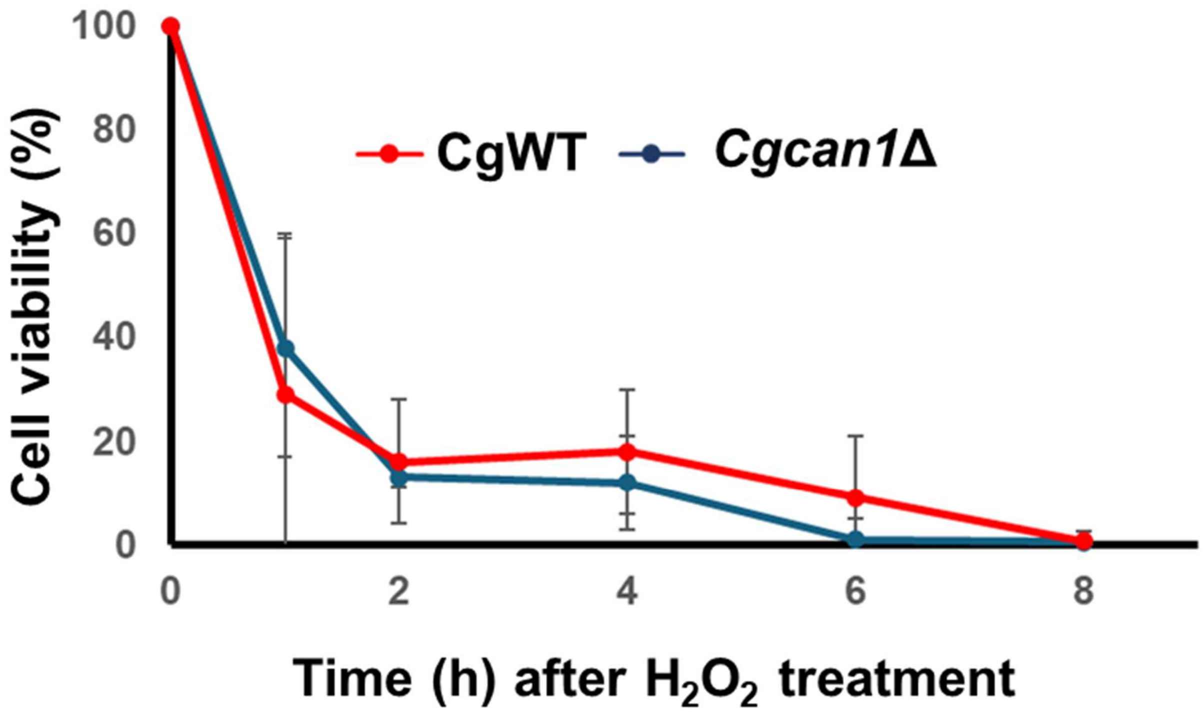

Supplementary Figure 4. Oxidative stress tolerance of cells in planktonic growth. Each strain was grown under shaking conditions, and cell viability was determined by measuring colony-forming units. Cells after treatment with 10 mM H_2O_2 were subjected to viability assessment at the indicated time points. Cell viability before H_2O_2 treatment was taken as 100. Data are presented as means ± SD (n=3), and statistical significance was determined by two-way ANOVA with Tukey's test.

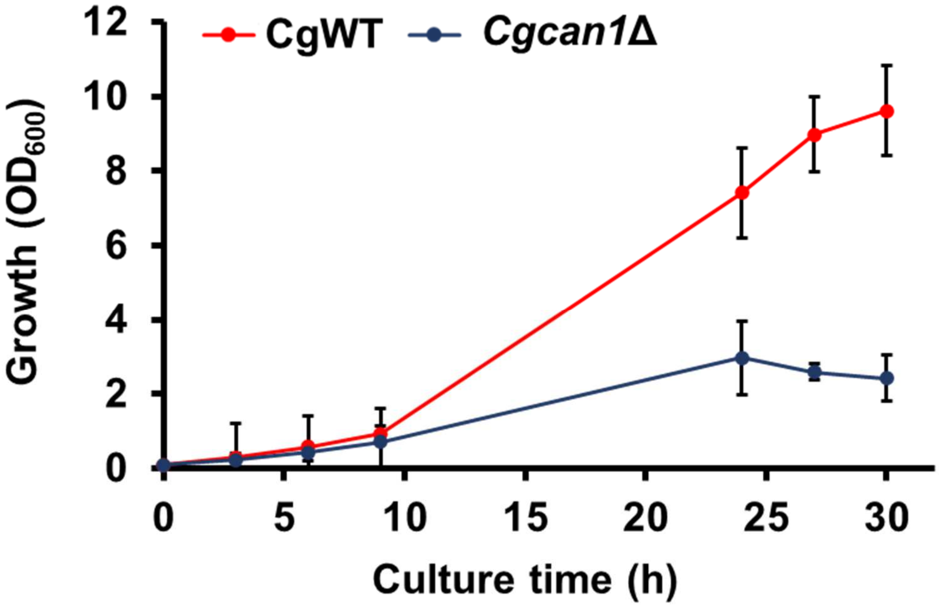

Supplementary Figure 5. Growth curve of *Cgcan1Δ*. Cell growth in the DMEM medium was determined at the indicated time points by measuring OD_{600}. Data are presented as means ± SD (n=3), and statistical significance was determined by two-way ANOVA with Tukey's test. *$p < 0.05$, vs. WT.

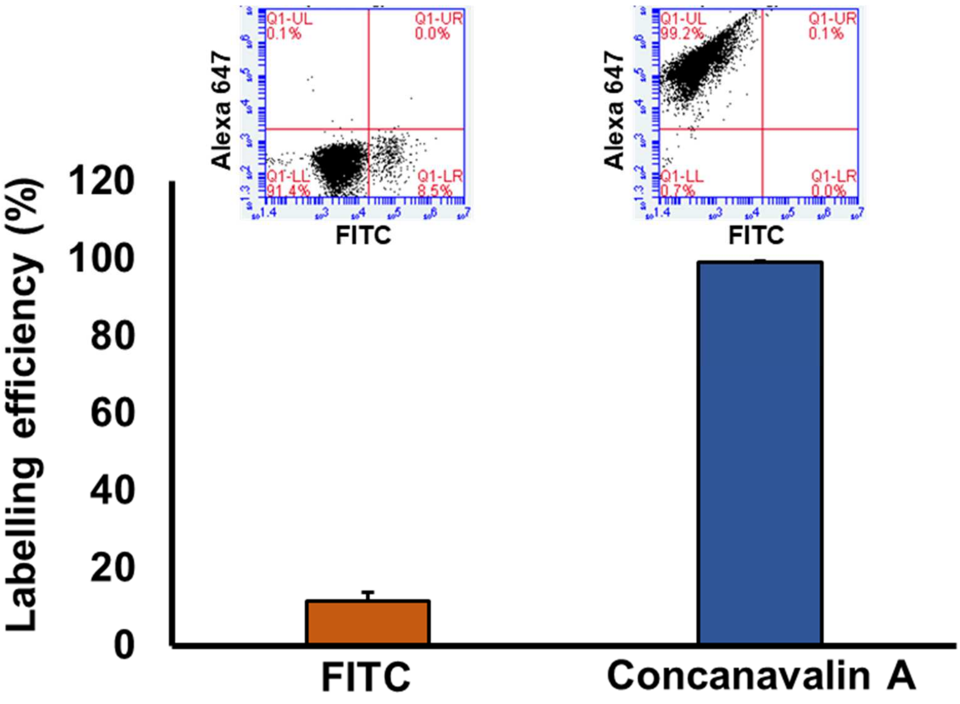

Supplementary Figure 6. Labeling efficiency of *C. glabrata* with FITC or concanavalin A. WT cells were treated with FITC or Alexa 647-conjugated concanavalin A (concanavalin A) and analyzed by flow cytometry. Upper photographs show the flow cytometry dot plot. The lower indicates the quantitation of the flow cytometry analysis (10,000 cells).

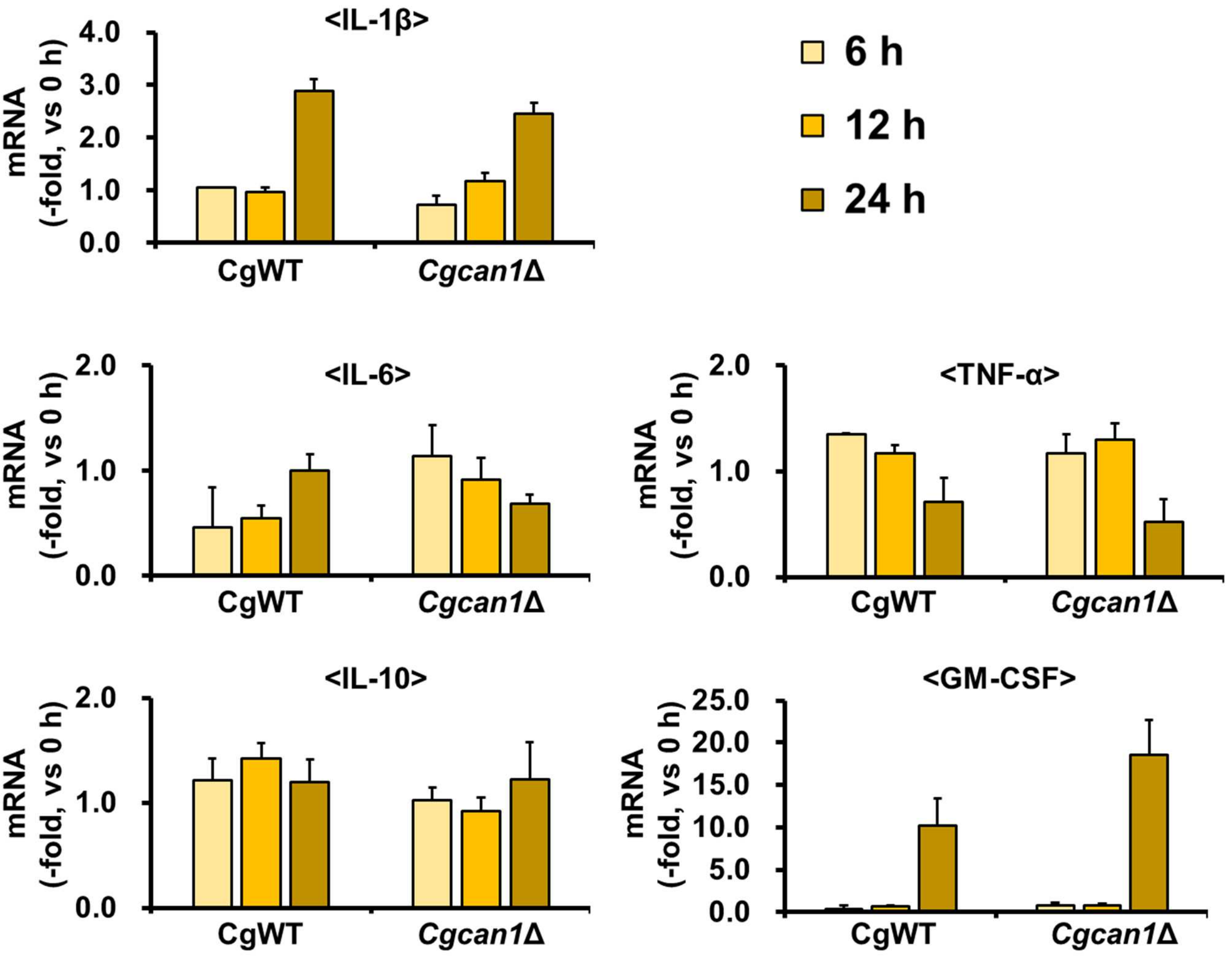

Supplementary Figure 7. Expression levels of cytokine genes in macrophage-like cells. Cytokine genes (*IL-1β*, *IL-6*, *TNF-α*, *IL-10*, and *GM-CSF*) were determined by qPCR at the indicated time points after infection. Data are presented as means ± SD ($n = 3$).
